# A rare case of important and recurrent abnormal uterine bleeding in a post partum woman caused by cavernous hemangioma: a case report and review of literature

**DOI:** 10.11604/pamj.2017.28.130.10084

**Published:** 2017-10-10

**Authors:** Kacou Edele Aka, Gninlgninrin Apollinaire Horo, Minata Fomba, Salif Kouyate, Abdoul Koffi Koffi, Seni Konan, Mohamed Fanny, Benjamin Effi, Mamourou Kone

**Affiliations:** 1 Department of Obstetrics and Gynecology, University Hospital of Yopougon, Abidjan, Ivory Coast; 2 Department of Pathological Anatomy, University Hospital of Treichville, Abidjan, Ivory Coast

**Keywords:** Hemangioma cavernous, uterine bleeding, post-partum, Africa

## Abstract

The cavernous hemangioma is a rare benign vascular tumor. About 50 cases of this disease were found in the literature over the last century and only 9 cases of cavernous hemangioma on the pregnant uterus were published it comes into cavernous or capillary form. The symptomatology is not unequivocal and when it occurs during pregnancy or postpartum, it causes life-threatening cataclysmic hemorrhage. Antenatal diagnosis is difficult and requires a multidisciplinary approach with pathologists, radiologists and gynecologists to avoid these complications or unnecessary hysterectomies. The diagnosis is histological. Hysterectomy is possible after failure of conservative treatment means. We report a rare case, a novel mixed cavernous hemangioma of the body associated with a capillary hemangioma of the cervix in a patient of 28 years 5th visors with recurrent genital bleeding in the postpartum period leading to a hysterectomy.

## Introduction

The cavernous hemangioma is a rare benign vascular tumor. About 50 cases of this disease were found in the literature over the last century and only 9 cases of cavernous hemangioma on the pregnant uterus have been published [1, 2], with the first reported case in the autopsy 1897. It can affect all the coats of the uterine wall, especially the myometrium. The cavernous hemangioma may be congenital, associated with an inherited bleeding (telangiectasia) or, acquired due to surgery, trophoblastic disease, pelvic inflammatory disease, cancer of the endometrium, or ingestion diethylstilbestrol [2]. Because of their small size and their asymptomatic nature, a majority of hemangiomas is discovered incidentally, but they may have abnormal vaginal bleeding.They are also associated with many gynecological and obstetric complications. This cycle disorder type spotting, dysfunctional uterine bleeding, impaired fertility and genital bleeding during pregnancy, childbirth and postpartum [3, 4]. Sometimes they may be in a fatal cataclysmic hemorrhage, requiring an emergency hysterectomy [2]. The diagnosis is histological examination of the operative part of hysterectomy [4]. We report a rare case, a novel cavernous hemangioma of the body associated with a capillary hemangioma of the cervix in a patient of 28 years multiparous with recurrent genital bleeding in the postpartum period leading to a hysterectomy.

## Patient and observation

**Figure 1:**
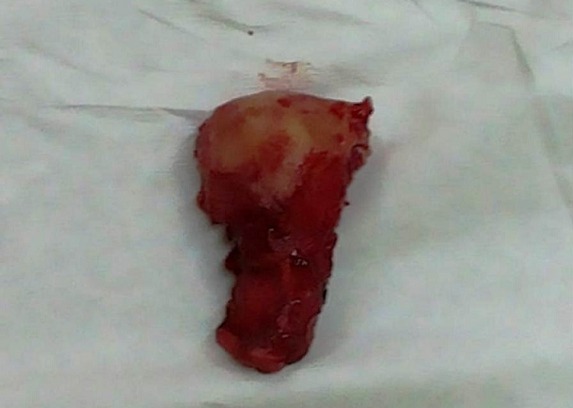
Macroscopic view of total hysterectomy specimen

**Figure 2:**
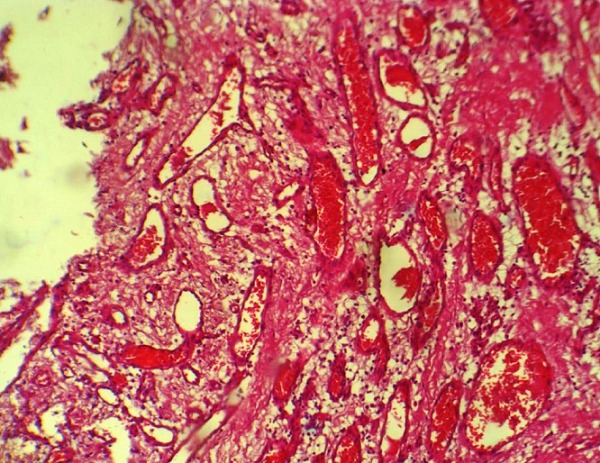
Cavernous hemangioma X 100 - vessels vary in size and dilated containing erythrocytes light

Mrs B.S. 28 years old multiparous that we had received 02 hours after a vaginal delivery of a newborn alive weighing 2750 grams in a peripheral maternity 3 km away. It was referred for postpartum hemorrhage. His medical history was featureless. On admission, she presented hypovolemic shock with blood pressure 76/44 mmHg, regular tachycardia at 136 beats per minute, polypnea to 39 cycles per minute. The obstetrical examination objectified an involuted uterus, an absence of vaginal and perineal cervical lesions, a fluid and red blood originating of endouterine. Furthermore, there was purpura. We therefore concluded a significant postpartum hemorrhage complicated by coagulopathy. Resuscitation measures were taken and an transfusion 1120 ml whole blood, red cell pellets 350 ml and 860 ml fresh frozen plasma have helped to management coagulopathy and anemia. Blood count at admission highlighted severe thrombocytopenia 23 0000 platelets and hemoglobin levels of 3.9 g/dl. The post natal care was also administered. After seven days of hospitalization, she was going after an amendment to the clinical symptoms and normal laboratory tests. Twenty five days later, she returned to obstetric emergencies for significant bleeding with severe anemia with hemoglobin 9 g/dL without signs of hypovolemic shock. The gynecological examination was normal. The balance sheet of the coagulation was normal including prothrombin to 92% and a normal activated partial thromboplastin time. The treatments were based on oxytocin, tranexamic acid, etamsylate and strict regular monitoring. Transvaginal ultrasound coupled Doppler was normal. In the absence of arterial embolization, she was going with ambulatory monitoring. Two weeks later she presented again rebleeding requiring hospitalization resulting in a total hysterectomy after conditioning of the patient (Figure 1). Histological examination of the surgical specimen objectified in the cervix and uterine body the presence of arterial vessels proliferation homes large and small caliber associated with tissue hemorrhage (Figure 2). The postoperative course was simple.

## Discussion

Hémangiomas are benign tumors [5] obtained are endothelial cells of blood vessels, or pericytes localized to the basal lamina of the endothelium of capillaries they surround. They appear in two forms: capillary and cavernous. Histologically, they are characterized by irregular anastomosing vascular spaces lined with endothelial cells, with intraluminal blood or thrombus. Cavernous hemangiomas are found in the skin, liver, kidney, breast, muscle, bowel wall, brain and bones. These are larger than the capillary type, sometimes in the form of a cave. Both types, capillary, cavernous, are found in the uterus. Capillary hemangiomas are generally limited to the endometrium and neck, while the cavernous form affects all coats of the uterus [6]. In our case, there is a mixed form, namely a cavernous hemangioma located at the myometrium associated to localized capillary hemangioma in the cervix. Its exact incidence is still difficult because of the very low number of cases reported about 50 cases including 9 on a century pregnant uterus. [2] This case is a rare case found in sub-Saharan Africa. The age of onset varies. The youngest patient found in the literature was 14 years [3] when the oldest was 70 years old [1, 3]. These lesions develop in two phases: the proliferative phase and the phase of involution. The cervical hemangioma may be congenital or acquired. The congenital hemangioma is very often associated with hereditary diseases such as Klippel-Trenaunay syndrome, hereditary hemorrhagic telangiectasia, tuberous sclerosis, Maffucci syndrome, and Kasabach-Merritt syndrome and endothelium in this case in the proliferation phase [7]. Acquired hemangioma is associated with both physical and hormonal changes [7]. Most of the reported cases are classified as acquired hemangiomas, and endothelial are generally in the involution. Several theories propose that hormones play a crucial role in the development of hemangiomas. Sun et al [8] proposed a model for the pathogenesis of the clinical database and laboratory that estrogen influences vasculogenesis and angiogenesis hemangiomas via an indirect route of angiogenic factors. Estrogen causes an increase in endothelial progenitor cells and angiogenic factors such as matrix metalloproteinase, vascular endothelial growth factor, nitric oxide, and other related factors. The hemangioma found in our patient would be acquired and due to hormonal influence.

The clinical symptomatology is variable. This condition can be asymptomatic or be in the form of abdominal pain, vaginal bleeding of variable abundance, anemia, fertility disorders and pregnancy-related complications [3, 7]. The amendments to during pregnancy can lead to risk of obstetric complications [3]. The most frequent complications are post-partum hemorrhage or disseminated intravascular coagulation (DIC). Hormonal changes and physical changes (increased blood volume) of the internal configuration of the uterus that occur during pregnancy or childbirth can altered pre-existing lesions and thus trigger DIC. The pathophysiology of this would be due to an accumulation of platelets trapped in the endothelium abnormally proliferative within hemangioma [3, 9]. Indeed, the patient presented a significant unexplained bleeding in the immediate postpartum uterus and retracted with a soft tissue integrity. The blood count made 02 hours after giving birth to his admission showed severe thrombocytopenia to 23,000 platelets. Computed tomography (CT) angiography, and magnetic resonance imaging (MRI) could guide us to the diagnosis. The ultrasound-guided biopsy can be performed for histologic diagnosis and may help to avoid unnecessary total hysterectomies, especially for women of childbearing age [3, 9]. The patient was financially limited and could only carry out a Transvaginal coupled to the non-contributory Doppler ultrasound. This benign tumor implies a conservative treatment with regular monitoring. Conservative measures used are local excision, cone biopsy, cauterization, radiation, suture ligation, cryotherapy, local and systemic steroids therapy, excision, laser photocoagulation and radiation therapy has been suggested as a possible treatment, but it could affect ovarian function [1, 2]. If hemangiomas are refractory to conservative treatment, hysterectomy may be considered. None of the above treatments had been undertaken in our patient. Definitive diagnosis is based on the final histological examination. This diagnosis has been retained after histological examination of the operative part of total hysterectomy. The differential diagnosis histologically is done with [10]: an adenomatoid tumor is a benign mesothelial tumor, lymphangioma, and arteriovenous malformation which is composed of a mass of arterial and venous vessels of different sizes, with the formation of fistulas them visible on ultrasound Doppler transvaginal coupled unlike hemangioma.

## Conclusion

The cervical hemangioma is a rare vascular tumor. It comes in cavernous or capillary form. Our case is interesting because into the case of a mixed form combining cavernous hemangioma capillary myometrium and cervix. The symptomatology is not unequivocal and when it occurs during pregnancy or postpartum, it causes life-threatening cataclysmic hemorrhage. The diagnosis is essentially histological. The management should be multidisciplinary with pathologists, radiologists and gynecologists avoid these complications or unnecessary hysterectomies.

## Competing interests

Authors declare no competing interests.
